# Third-Degree Hindpaw Burn Injury Induced Apoptosis of Lumbar Spinal Cord Ventral Horn Motor Neurons and Sciatic Nerve and Muscle Atrophy in Rats

**DOI:** 10.1155/2015/372819

**Published:** 2015-01-28

**Authors:** Sheng-Hua Wu, Shu-Hung Huang, Kuang-I Cheng, Chee-Yin Chai, Jwu-Lai Yeh, Tai-Cheng Wu, Yi-Chiang Hsu, Aij-Lie Kwan

**Affiliations:** ^1^Graduate Institute of Medicine, College of Medicine, Kaohsiung Medical University, Kaohsiung 80708, Taiwan; ^2^Department of Anesthesia, Kaohsiung Medical University Hospital, Kaohsiung Medical University, Kaohsiung 80708, Taiwan; ^3^Kaohsiung Municipal Hsiao-Kang Hospital, Kaohsiung Medical University, Kaohsiung 80708, Taiwan; ^4^Department of Surgery, Faculty of Medicine, College of Medicine, Kaohsiung Medical University, Kaohsiung 80708, Taiwan; ^5^Division of Plastic Surgery, Department of Surgery, Kaohsiung Medical University Hospital, Kaohsiung Medical University, Kaohsiung 80708, Taiwan; ^6^Center for Stem Cell Research, Kaohsiung Medical University, Kaohsiung 80708, Taiwan; ^7^Department of Pathology, Kaohsiung Medical University Hospital, Kaohsiung 80708, Taiwan; ^8^Department and Graduate Institute of Pharmacology, College of Medicine, Kaohsiung Medical University, Kaohsiung 80708, Taiwan; ^9^Division of Neurology, Department of Internal Medicine, Kaohsiung Armed Forces General Hospital, Kaohsiung 80284, Taiwan; ^10^Graduate Institute of Medical Science, College of Health Sciences, Chang Jung Christian University, No. 1, Changda Road, Gueiren District, Tainan City 71101, Taiwan; ^11^Innovative Research Center of Medicine, College of Health Sciences, Chang Jung Christian University, No. 1, Changda Road, Gueiren District, Tainan City 71101, Taiwan; ^12^Division of Neurosurgery, Department of Surgery, Kaohsiung Medical University Hospital, 100 Tzyou 1st Road, Kaohsiung 80708, Taiwan

## Abstract

*Background*. Severe burns result in hypercatabolic state and concomitant muscle atrophy that persists for several months, thereby limiting patient recovery. However, the effects of burns on the corresponding spinal dermatome remain unknown. This study aimed to investigate whether burns induce apoptosis of spinal cord ventral horn motor neurons (VHMNs) and consequently cause skeletal muscle wasting. *Methods*. Third-degree hindpaw burn injury with 1% total body surface area (TBSA) rats were euthanized 4 and 8 weeks after burn injury. The apoptosis profiles in the ventral horns of the lumbar spinal cords, sciatic nerves, and gastrocnemius muscles were examined. The Schwann cells in the sciatic nerve were marked with S100. The gastrocnemius muscles were harvested to measure the denervation atrophy. *Result*. The VHMNs apoptosis in the spinal cord was observed after inducing third-degree burns in the hindpaw. The S100 and TUNEL double-positive cells in the sciatic nerve increased significantly after the burn injury. Gastrocnemius muscle apoptosis and denervation atrophy area increased significantly after the burn injury. *Conclusion*. Local hindpaw burn induces apoptosis in VHMNs and Schwann cells in sciatic nerve, which causes corresponding gastrocnemius muscle denervation atrophy. Our results provided an animal model to evaluate burn-induced muscle wasting, and elucidate the underlying mechanisms.

## 1. Introduction

Severe burns are typically followed by a hypermetabolic state with increased proteolysis and lipolysis [1], which persists for at least 9 to 12 months after burns even after complete wound closure [2], thereby resulting in considerable erosion of the lean body mass, muscle weakness, and diminished capacity for complete rehabilitation. The current standard of care during recovery for severe burns comprises administration of nutrients and aggressive rehabilitation; however, the results are just partial, with incomplete restoration of previous musculature and strength [3–6].

By contrast, major burn injuries, including both electrical and thermal burn injuries, are associated with peripheral neuropathies [7, 8]. These neuropathies include mononeuropathy, generalized peripheral polyneuropathy, and axonal neuropathy of both motor and sensory nerves [8, 9]. In addition, burn-induced nerve conduction deficits have been observed in 11% to 29% of patients with major burns, particularly in older or critically ill patients [10]. These neuropathies cause skeletal muscle weakness and wasting [11], the underlying etiology of which remains unclear [12].

Higashimori et al. used an animal model of 20% total body surface area (TBSA) full-thickness burn injury induced on the back of mice to evaluate whether systemic burns affected the motor neuron morphology in the fifth lumbar vertebra (L5); although no morphological changes were observed 14 days after the dermal burn injury, a significant reduction in the motor conduction velocities of the large myelinated motor nerves was noted [9]. Few studies have focused on the effects of burn injuries on the viability of the ventral horn motor neurons (VHMNs) at the corresponding dermatome and muscle mass loss.

Several in vitro and in vivo studies have documented the burn-induced apoptotic death of cardiomyocytes and skeletal muscles [13–15]. Apoptosis and cell cycle arrest seem to be the common mechanisms of cell death [16]. Two major apoptotic pathways exist: the death receptor and the mitochondrial pathways [17]. Multiple apoptotic stimuli trigger the activation of proteases called caspases, which, in turn, initiate and execute the apoptotic process [18]. The activation of the executorial caspases seems indispensable for realization of the apoptotic program [19]. Subsequently, the truncated Bcl-2 interacting domain migrates to the mitochondria, which leads to the loss of permeabilization of the mitochondrial membrane, cytochrome *c* release, and activation of the initiator caspase-9 [20]. Caspase-9- and caspase-3-like activity and plasma membrane disintegration served as measures for early apoptosis, whereas nuclear fragmentation served as an indicator of late apoptotic events [21].

Based on the above understanding, we hypothesize that local third-degree burn injuries will cause apoptotic death of VHMNs of the corresponding spinal dermatome, which will lead to denervation atrophy of the affected muscle, thereby causing muscle atrophy and weakness. We designed experiments to test this hypothesis by inducing third-degree burns in the hindpaw, and the animals were euthanized at 4 and 8 weeks after the injury. Apoptosis of the corresponding spinal cord VHMNs was determined by caspase-9, caspase-3, and TUNEL assay; the sciatic nerve was assessed using S100 and TUNEL assay; and the gastrocnemius muscle was examined by hematoxylin-eosin staining. Our results revealed that third-degree burns induced VHMN apoptosis and consequently caused muscle denervation atrophy. These findings suggest that third-degree burn injuries cause VHMN apoptosis of the corresponding spinal dermatome in patients suffering from such injuries; however, further research is warranted to discover clinical strategies to prevent such postburn complications.

## 2. Methods

### 2.1. Experimental Design

**Figure 1:**
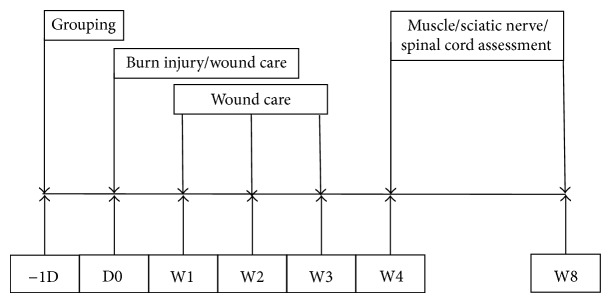
Flow chart of the study plan.

Male Sprague-Dawley rats, weighing 180 to 200 g, were fed with standard lab rodent chow and water, available* ad libitum*, and housed individually. The animal study protocols were approved by the Institutional Animal Care and Use Committee of the Kaohsiung Medical University (Approval number 102173).  Figure 1 presents a flow chart depicting the study design. The rats were divided into 3 treatment groups (*n* = 6 for each group) as follows: Group A: sham burn injury, Group B: euthanized 4 weeks after burn injury, and Group C: euthanized 8 weeks after burn injury.

### 2.2. Full-Thickness Burn Injury Model

The animals were anesthetized through a subcutaneous injection of Zoletil 50 (50 *μ*g/g; Virbac Laboratory, Carros, France). Full-thickness burn injuries were induced in the hindpaw model, as reported previously [22]. In brief, the hindpaw skin was placed on a metal block at 75 ± 0.5°C and a 100 g weight was placed on the paw to maintain constant contact for 10 seconds. After inducing the burn injury, pain control and silver sulfadiazine cream was administered to the rat until the wound healed.

### 2.3. Measurement of Apoptosis in the Spinal Cord and Sciatic Nerve

The rats were anesthetized with Zoletil 50 and perfused with 200 mL of heparinized saline and 4% paraformaldehyde in 0.1 M phosphate buffered saline (PBS). The L3 to L5 segments and the right sciatic nerve were removed and postfixed overnight in 4% paraformaldehyde in 0.1 M PBS at 4°C before being transferred into a 30% sucrose solution. The spinal cord was embedded in an optimal cutting temperature (OCT) compound to prepare frozen sections cut into 16 *μ*m thick slices. The sciatic nerve was embedded in 4 *μ*m thick slices of paraffin. Apoptotic cell death was detected using the TUNEL assay according to the manufacturer's suggestions (Millipore, ApopTag fluorescein in situ apoptosis detection kit S7110). The samples were fixed using ethanol and acetone (9 : 1) at 4°C for 5 minutes and were then rinsed 3 times with PBS for 5 minutes each, followed by an addition of protease K (20 *µ*g/mL for 15 min). The samples were rinsed 3 times with PBS for 5 minutes each and immersed in an equilibration buffer for 2 minutes. After that, sections were incubated in a terminal deoxynucleotidyl transferase enzyme mixture for 1 hour at 37°C in a humidified chamber. Subsequently, the sections were rinsed in a stop/wash buffer for 15 minutes and 3 times in PBS for 5 minutes each and then incubated (avoiding light) with antidigoxigenin fluorescein for 30 minutes for detection. After the sections were rinsed 3 times in PBS for 5 minutes each and incubated with 5% normal goat serum for 1 hour, the sections were incubated overnight with NeuN primary antibody (1 : 1000, Merck Millipore, Bedford, MA) or S100 (1 : 200, Abcam, Cambridge, UK) at 4°C. Subsequently, the sections were incubated with Cy3-conjugated anti-mouse IgG secondary antibody (Merck Millipore, Bedford, MA) at room temperature for an additional 1 hour, rinsed 3 times with PBS for 5 minutes each, and then mounted with a mounting medium containing 4,6-diamidino-2-phenylindole (DAPI). The sections were mounted on glass slides and covered. The TUNEL/NeuN/S100-positive cells were counted in 3 fields (400x) for each section of each rat in each group. The images were recorded using a Leica DMI6000 inverted microscope.

### 2.4. Immunofluorescence Stain Detection of Caspase-3 and Caspase-9 in the Spinal Cord

The rats were anesthetized with Zoletil 50 and perfused with 200 mL of heparinized saline and 4% paraformaldehyde in 0.1 M PBS. The L3 to L5 segments were removed and were soaking overnight in 4% paraformaldehyde in 0.1 M PBS at 4°C before being transferred into a 30% sucrose solution. The spinal cord was embedded in an OCT compound to prepare frozen sections cut into 16 *μ*m thick slices. The sections were incubated in 5% normal goat serum for 1 hour at room temperature and then incubated overnight with cleaved caspase-3 (1 : 200; Cell Signaling, Beverly, MA, USA), caspase-9 (1 : 200; Abcam, Cambridge, UK), and NeuN primary antibody (1 : 1000; Merck Millipore, Bedford, MA) at 4°C. The sections were washed with PBS before being incubated with goat anti-mouse 488 and Cy3-conjugated goat anti-rabbit IgG secondary antibodies for 2 hours at room temperature. The nuclear-staining agent DAPI was added as well. After a final wash with PBS, the sections were mounted on glass slides, air-dried, and covered.

### 2.5. Histology of Gastrocnemius Muscle

The animals were anesthetized with Zoletil 50 (50 *μ*g/g; Virbac Laboratory, Carros, France). The gastrocnemius muscles were harvested and placed in liquid nitrogen. The tissues were embedded in an OCT compound, and sections were obtained perpendicular to the muscle fibers with 10 *μ*m thick slides. Hematoxylin-eosin staining was performed according to routine histologic protocols. The muscle sections were observed by microscopy (Nikon, Ti-U, USA). The areas of muscle were counted in 3 fields (200x) for each section of each rat in each group and processed for statistical analysis.

### 2.6. Western Blot

For protein extraction, the gastrocnemius muscle was homogenized in a protein lysis buffer in the presence of a protease inhibitor and then incubated. The samples were centrifuged at 13 000 ×RPM at 4°C for 30 minutes. The total protein content of the supernatants was determined using Bradford R250. For Western blot analysis, equal amounts of the total protein content were separated by sodium dodecyl-sulfate polyacrylamide gel electrophoresis (SDS-PAGE; 15%) and transferred onto membranes. After blocking for 1 hour with 5% nonfat milk, the membranes were incubated overnight at 4°C with primary antibody light chain-3B (LC3B) (cell signaling, 1 : 1000) and *β*-actin (Sigma, 1 : 20 000). After several washes with TTBS, a secondary antibody, goat anti-rabbit-HRP, and goat anti-mouse-HRP were applied for 1 hour at room temperature. The peroxidase activity was visualized using the ECL Western Blotting Detection kit and the Bio-Rad ChemiDoc XRS system. The Western blots were quantified and plotted according to the average band intensity of 3 independent experiments, as determined using the ImageJ software.

### 2.7. Measurement of TNF-*α* and IFN-*γ* in Blood Samples

Anesthetizing the animals with Zoletil 50 at 1 day and 4 and 8 weeks after the burn injuries collected serum samples. Serum TNF-*α* and IFN-*γ* levels were measured using enzyme-linked immunosorbent assay kits for the quantitative detection of TNF-*α* and IFN-*γ* (Invitrogen, Camarillo, CA). The reference range for the enzyme-linked immunosorbent assay kit was 0 to 750 (TNF-*α*) and 0 to 1400 (IFN-*γ*) pg/mL. When the value of a sample fell outside the aforementioned reference ranges, the sample was reanalyzed at a higher dilution. All the measurements were recorded in triplicate, and their mean values were calculated.

### 2.8. Statistical Analysis

Intergroup comparisons were performed using one-way analysis of variance (ANOVA). The mean and standard deviation values were calculated according to the numerical data, as presented in the figures and figure legends. Bar graphs were used to represent the means, whereas error bars were used to represent the standard deviations. A *P* value of <.05 was considered statistically significant.

## 3. Results

### 3.1. Full-Thickness Burn Injury Induced Apoptosis in Lumbar Spinal Cord Ventral Horn Motor Neurons

To measure the apoptotic event of the spinal cord after the full-thickness burn injury, the lumbar spinal cord segments were harvested and evaluated by caspase-3, caspase-9, and TUNEL assay. For double immunofluorescence staining, the neurons in the spinal cord were labeled with the NeuN stain. Significant differences were observed between Groups A and B with regard to caspase-3/NeuN, caspase-9/NeuN, and TUNEL/NeuN (*P* = .032, .036, and .046, resp.).

**Figure 2:**
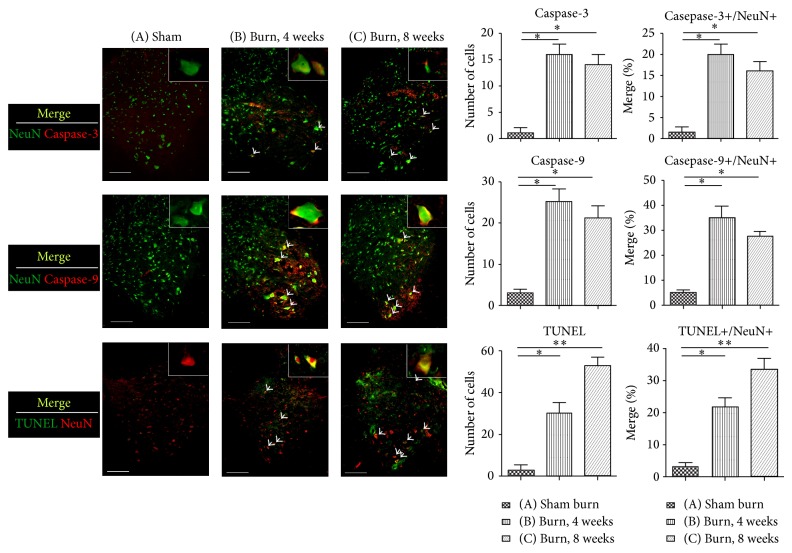
Double immunofluorescence staining of caspase-3, caspase-9, TUNEL assay, and a neuronal cell marker, NeuN, in the spinal cord ventral horns of rats from different groups. Tissue samples were detected using antibodies against NeuN, caspase-3, caspase-9, and TUNEL staining for apoptosis. The merge images depict apoptosis of the motor neurons in the spinal cord ventral horns of the rats. The arrowheads indicate double-positive neurons. The bar chart depicts the number of positive cells (caspase-9, caspase-3, and TUNEL) and the proportion of double labeling cells (caspase-9/NeuN, caspase-3/NeuN, and TUNEL/NeuN), showing that the apoptotic events were triggered by the third-degree burn injuries in the hindpaw skin. Histogram represents mean ± SEM (^*^ *P* < .05, ^**^ *P* < .01; scale bar = 200 *μ*m).

These values for Group C were as follows: *P* = .022, .018, and .0097 (Figure 2, ^*^ *P* < .05, ^**^ *P* < .001 versus Group A). These results indicated that spinal cord VHMNs underwent apoptosis after burn injury, and this phenomenon persisted for up to 8 weeks.

### 3.2. Full-Thickness Burn Injury Induced Apoptosis in Sciatic Nerve

**Figure 3:**
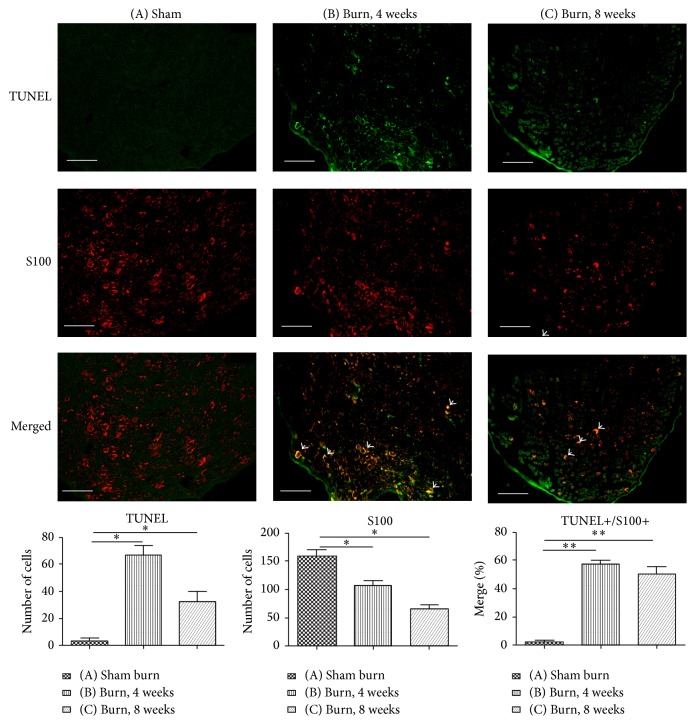
Double immunofluorescence staining of TUNEL and the Schwann cell marker S100 in the ipsilateral sciatic nerve in different groups. The number of TUNEL+ cells in the sciatic nerve increased significantly after the burn injury at 4 and 8 weeks compared with that in the sham burn group. Immunolabeling of S100 revealed that the number of Schwann cells in the sciatic nerve decreased significantly at 4 and 8 weeks after the burn injury compared with that in the sham burn group. The arrowheads indicate the TUNEL/S100 double-positive cells. After the burn injury, the percentage of merge cells in TUNEL+/S100+ increased significantly in the sciatic nerve. Data are plotted as mean ± SEM. ^*^ *P* < .05, ^**^ *P* < .01 versus the sham burn group.

The Schwann cell is a glial cell that surrounds the nerve fiber in the peripheral nervous system (PNS) and forms myelin sheaths to support the PNS axons. The specific stain for Schwann cells is S100 [23]. To determine sciatic nerve apoptosis, we used double immunofluorescence staining with S100 and TUNEL assay (Figure 3). The results showed that the number of S100+ cells decreased significantly at 4 and 8 weeks after the burn injury (*P* = .025 and *P* = .013, resp.), whereas the proportion of merge cells increased significantly at 4 and 8 weeks after the burn injury (*P* = .009 and *P* = .007, resp.). These results may indicate that third-degree burns cause apoptosis of the Schwann cells of the sciatic nerve, which persists up to 8 weeks.

### 3.3. Gastrocnemius Muscle Atrophy and Apoptosis following Burn Injury

**Figure 4:**
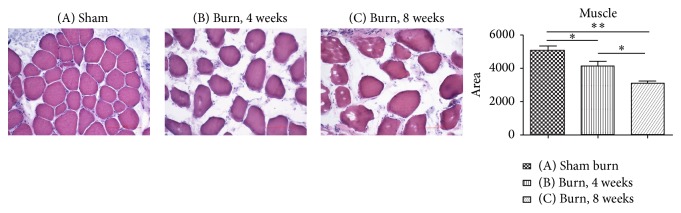
Hematoxylin-eosin staining of the gastrocnemius muscles was performed. The muscle fiber areas were recorded in the different groups. The area of the muscle fiber decreased significantly at 4 and 8 weeks after the burn injury, with a progressive course. The data are plotted according to the areas of muscle fiber as mean ± SEM. ^*^ *P* < .05 versus the sham burn group.

Histologic examination of the gastrocnemius muscle samples was performed at 4 and 8 weeks after the burn injury, and the areas of the muscles were recorded. The results revealed a significant decrease in the areas in Groups B and C compared to that in Group A (*P* = .043 and *P* = .0008) (Figure 4). These muscle fibers are caused by muscle denervation [24].

**Figure 5:**
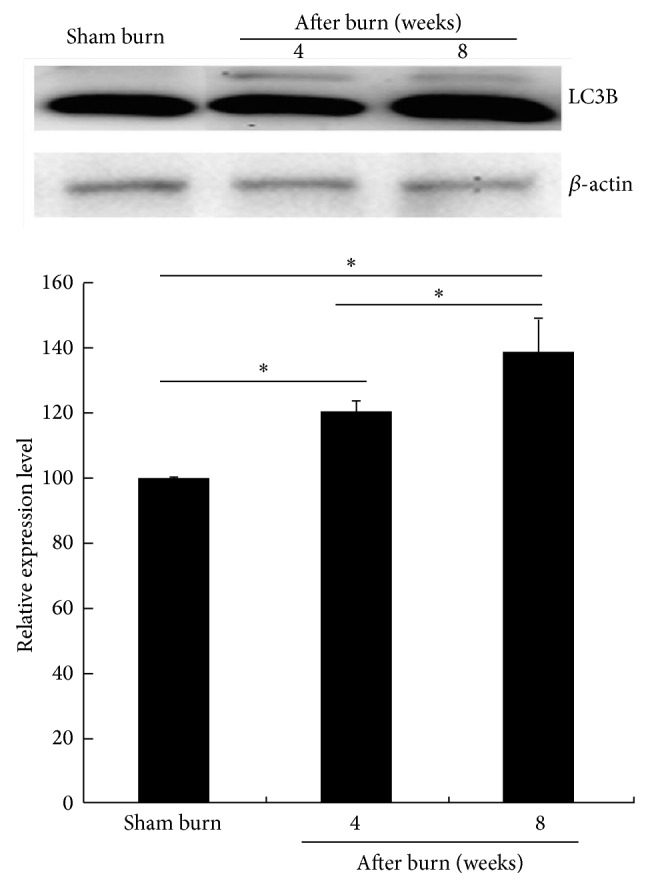
Immunoblotting showing the protein expression levels of LC3B in the gastrocnemius muscle. The LC3B protein expression levels recorded for each group tested are shown as a bar chart of the relative ratios normalized with *β*-actin. ^*^ *P* < .05 compared with sham burn for each group. Representative Western blot (above) and densitometry quantification (below).

To evaluate the LC3B protein expression on the gastrocnemius muscles at 4 and 8 weeks after the burn injury, samples prepared from the ipsilateral limb were immunoblotted. The LC3B expression levels in Groups B and C were significantly higher than those in Group A (Figure 5). LC3B is an autophagic marker, which accounts for the protein breakdown mediated by the autophagic or lysosomal pathways [25]. Our results revealed that the LC3B expression in the muscles increased significantly at 4 and 8 weeks after the burn injury compared to that in the sham burn group (*P* = .036 and *P* = .026, resp.).

Based on these results, we suggested that third-degree burns could induce excessive autophagy activation, which contributes to muscle loss and apoptosis and is characterized by muscle denervation atrophy.

### 3.4. No Changes in Serum TNF-*α* and INF-*γ* Levels at 1 Day and 4 and 8 Weeks after Burn Injury

The serum TNF-*α* and INF-*γ* levels were compared after burn injury using serum samples collected at 1 day and 4 and 8 weeks after the burn injury. However, no significant differences were observed in the serum TNF-*α* and INF-*γ* levels among the 3 groups.

## 4. Discussion

Hypercatabolism following major burn injuries causes muscle wasting, as demonstrated by most studies focusing on the anabolic and catabolic processes or morphological changes at the muscle level [1, 2, 6, 26–28]. Wu et al. investigated the activation of the satellite cells following burn injuries and Merritt et al. focused on the inflammatory and protein metabolism signaling responses of the skeletal muscles after burn injuries [29, 30]. In addition, peripheral neuropathies, such as polyneuropathy and axonal neuropathy, have been observed following major burn injuries [7, 8]. However, few studies have focused on the effects of burn-induced injuries on spinal cord VHMNs. This paper presents a straightforward and reproducible model of apoptosis of spinal cord VHMNs following third-degree burn injuries. Reportedly, third-degree burns on the hindpaw (dermatomes L3–L5) [31, 32] induce apoptosis of the spinal cord VHMNs at the corresponding dermatomes and consequently cause gastrocnemius muscle wasting (innervated by L4 and L5) [33].

Higashimori et al. established a burn animal model, wherein the burn is induced over the dorsum from the base of the ears to the tail for approximately 20% of the TBSA, and evaluated nerve injury after burns [7, 9]. In these studies, the motor nerve conduction velocity of the tibial nerve remained markedly reduced even at 35 days after the burn injury [7], and the back dermatome including L1-S3 and sciatic nerve arises from L4 through S3. The myelin sheath is formed by the Schwann cells, which encircle the axon and can increase the conduction velocity. Tamam et al. examined 47 burn patients with peripheral neuropathy and reported axonotmesis in 63.8% of the patients and demyelination in 29.8% as the form of neuropathy [8]. Our study results revealed apoptosis of the Schwann cells of the sciatic nerve, with a decrease in the number of cells after the burn injury. We can speculate that the decreased motor nerve conduction velocity in the tibial nerve after the injury may be responsible for the Schwann cell apoptosis as well as the decrease in the cell number.

The development of axonotmesis neuropathy and Wallerian degeneration after burn injuries has been well documented. When a nerve gets injured, the axonal stump becomes swollen and edematous during the initial few days; following this, Wallerian degeneration commences, which involves axonal and myelin disintegration in the antegrade and retrograde directions [34]. Whiteside and Munglani demonstrate that a chronic constriction injury to the sciatic nerve caused apoptotic cell death in the ipsilateral spinal cord of the rat 14 days after injury [35]. Abe et al. demonstrated that an avulsion injury to the sciatic nerve caused apoptosis of the spinal cord VHMNs after the injury, with p53 activation and Bax upregulation in a rat model with chronic constriction injury of the sciatic nerve [36]; in addition, TUNEL-positive or apoptotic cells were detected in the ipsilateral dorsal root ganglion at 30 days after nerve injury [37]. We speculate that the third-degree hindpaw burn injury results in dermal neuron receptor injury and intrinsic muscle damage, leading to retrograde Wallerian degeneration through the sciatic nerve (both sensory and motor neuron fibers), which eventually injures the sensory neurons of the dorsal root ganglion and causes spinal cord VHMNs apoptosis. Our data showed that the decrease in the number of Schwann cells in the sciatic nerve and apoptosis after the burn injury led to the apoptotic cell death of the spinal cord VHMNs and dorsal horn neurons.

Loss of muscle mass occurs in several conditions, such as denervation, inactivity, cancer, and diabetes [38]. Autophagosomes have been identified in most myopathic and dystrophic diseases; LC3 is the mammalian homology of the yeast Atg8 gene and is critical for membrane commitment and growth. Knockdown of the critical gene LC3 by RNAi partially prevents FoxO3-medicated muscle loss in adult CD1 mice [39]. In the present study, elevated expression of the microtubule-associated protein-1 LC3B was observed in the gastrocnemius muscles of a mammal. The conversion of LC3B from its cleaved form to a conjugated form is regarded as a crucial step in the induction of autophagosome formation [40]. Subsequently, autophagosomes fuse with lysosomes to form autolysosomes [41], which digest the protein contents of the muscle fibers by proteases and other lysosomal hydrolases, thereby resulting in muscle atrophy.

The inflammatory responses after burn injuries include the generation of abacterial cytokines; several studies have demonstrated the production of proinflammatory cytokines, such as TNF-*α*, IL-1*β*, and IL-6 [42, 43]. However, the minimal amount of TBAS that induces a significant increase in the TNF-*α*, IL-1*β*, and IL-6 expression of the serum remains unclear. Yeh et al. reported that patients with burns >50% TBSA exhibited significant differences in their serum IL-10 levels between 5 and 20 hours after the injury [44]. Kim et al. evaluated 25 patients with 15% to 30% TBSA burn injuries and reported that although the highest IL-6, IL-8, and IL-10 levels were recorded 1 day after the injury, no significant increase was observed in the TNF-*α* levels [45]. In an animal model, 30% TBSA full-thickness rat burn model showed significantly increased TNF-*α*, IL-1*β*, and IL-6 levels at 3 hours after the burn injury [42]. Reportedly, circulating cytokines, such as IL-18, granulocyte colony stimulating factor, IL-10, IL-12, and IL-1*α*, were significantly elevated during the initial 24 hours after injury in rats with 20% TBSA burn injuries [46]. In addition, the development of dysfunction of multiple organs (e.g., liver, skeletal muscle, heart, and central nervous system) following severe burn injuries has been reported in both animals and humans; this comorbidity is believed to be caused by the increased apoptotic cell death and enhanced production of inflammatory cytokines such as TNF-*α*, IL-1*β*, and IL-6 [47]. However, in our model, no significant elevation was observed in the serum cytokine levels after the burn injury; this is attributed to the small area of burn, which exerted no systemic inflammatory effects. Therefore, 1% TBSA burn injuries in the hindpaw can cause spinal cord VHMN apoptosis but do not exert any systemic effects inducing inflammatory cytokine production.

The strength of this model is that we demonstrate that a small area burn (1% TBSA) can cause correlated dermatome spinal cord VHMNs apoptosis followed by muscle wasting. This could be applied to studying the mechanisms of burn-induced apoptosis and workout treatment agents for burn-induced muscle wasting. Nevertheless, this model has several limitations including the following: (a) the duration of spinal cord VHMNs apoptosis is unknown; (b) area of burn that will trigger the apoptosis is not documented; (c) other injuries (skin excision or crashing injury) that might also cause spinal cord VHMNs apoptosis are not verified.

In conclusion, this study demonstrates that burn-induced muscle atrophy may be caused by apoptosis of the VHMNs of the corresponding spinal dermatome. Our results suggest that when a full-thickness burn injury is induced, the VHMNs of the corresponding spinal dermatome as well as the Schwann cells of the corresponding nerve undergo apoptosis, which persists for up to 8 weeks. However, further research is warranted to determine appropriate clinical strategies to prevent such burn sequela and to further elucidate the underlying mechanisms or pathways.
